# Interaction of iron status with single nucleotide polymorphisms on incidence of type 2 diabetes

**DOI:** 10.1371/journal.pone.0175681

**Published:** 2017-04-13

**Authors:** Jihye Kim, Mi Kyung Kim, Sukyoung Jung, Ji Eun Lim, Myung-Hee Shin, Yeon-Jung Kim, Bermseok Oh

**Affiliations:** 1 Department of Preventive Medicine, College of Medicine, Hanyang University, Seoul, South Korea; 2 Institute for Health and Society, Hanyang University, Seoul, South Korea; 3 Department of Biomedical Engineering, School of Medicine, Kyung Hee University, Seoul, South Korea; 4 Social and Preventive Medicine, Sungkyunkwan University School of Medicine, Seoul, South Korea; 5 Division of Structural and Functional Genomics, Center for Genome Science, Korea National Institute of Health, Chungcheongbuk-do, Korea; University of Rochester, UNITED STATES

## Abstract

The objective of this study is to find single nucleotide polymorphisms (SNPs) associated with a risk of Type 2 diabetes (T2D) in Korean adults and to investigate the longitudinal association between these SNPs and T2D and the interaction effects of iron intake and average hemoglobin level. Data from the KoGES_Ansan and Ansung Study were used. Gene-iron interaction analysis was conducted using a two-step approach. To select candidate SNPs associated with T2D, a total of 7,935 adults at baseline were included in genome-wide association analysis (step one). After excluding T2D prevalent cases, prospective analyses were conducted with 7,024 adults aged 40–69 (step two). The association of selected SNPs and iron status with T2D and their interaction were determined using a Cox proportional hazard model. A total of 3 SNPs [rs9465871 (CDKAL1), rs10761745 (JMJD1C), and rs163177 (KCNQ1)] were selected as candidate SNPs related to T2D. Among them, rs10761745 (JMJD1C) and rs163177 (KCNQ1) were prospectively associated with T2D. High iron intake was also prospectively associated with the risk of T2D after adjusting for covariates. Average hemoglobin level was positively associated with T2D after adjusting for covariates in women. We also found significant interaction effects between rs10761745 (JMJD1C) and average hemoglobin levels on the risk of T2D among women with normal inflammation and without anemia at baseline. In conclusion, KCNQ1 and JMJD1C may prospectively contribute to the risk of T2D incidence among adults over the age of 40 and JMJD1C, but CDKAL1 may not, and iron status may interactively contribute to T2D incidence in women.

## Introduction

Diabetes is a huge and growing problem. The worldwide prevalence of diabetes in those aged 20–79 years was estimated to be 8.3% in 2011[1], including approximately 12.4% of Koreans aged ≥ 30 years [2]. It has been estimated that the number of diabetic patients worldwide will reach 439 million adults by 2030, primarily due to population growth, lifestyle, and demographic changes [3].

Iron is a vital nutrient for humans and is a cofactor for various metabolic functions. However, excessive iron levels helps produce and amplify free radicals, which can lead to tissue damage [4]. In particular, because of inefficient hydrogen peroxide-inactivating enzymes, pancreatic beta cells are susceptible to oxidative stress [5]. With the exception of direct tissue damage, elevated oxidative stress may directly lead to increased blood glucose levels [6]. Additionally, increased heme iron intake is significantly associated with a greater risk of Type 2 diabetes (T2D) [7]. There is a positive relationship between an increased risk of T2D and serum ferritin, an indicator of total body iron stores [7, 8]. Additionally, higher hemoglobin level is also associated with a high risk of T2D in women [9].

Hepcidin, a peptide hormone synthesized by hepatocytes, is a key modulator of dietary iron absorption and iron release from macrophages [6], and increasing body iron stores induce hepcidin synthesis. Production of hepcidin is also induced by infection and inflammation, which result in decreased iron absorption and iron release from macrophages [10]. Conversely, anemia leads to a decrease in hepcidin production, thereby eliminating the inhibitory effect on intestinal iron absorption and release from macrophages [10]. Thus, inflammatory status and anemia need to be considered in epidemiologic studies regarding iron status.

Environmental, genetic factors, and their interactions contribute to the development of T2D [11]. Heritability for T2D was estimated to be about 26% [12]. Interactions between genetic and environmental factors are likely to be important in the remaining variance [13], but such studies analyzing SNPs and dietary iron intake are sparse. Previously, one study suggested a potential interaction between hemochromatosis (HFE), a major regulator of iron homeostasis, and heme iron intake [14]. However, another study did not find any new interactions between single nucleotide polymorphisms (SNPs) in the heme iron metabolic pathway and heme iron intake [15]. Currently, it is unclear whether iron levels interact with SNPs on the risk of T2D.

To the best of our knowledge, there have been no studies investigating the interaction effects between iron intake and gene polymorphisms in the Asian population. Elucidating gene-iron interactions could enhance our understanding of the development of T2D and lead to effective personalized prevention of this disease. Therefore, the objectives of this study were as follows: 1) to find new SNPs associated with the risk of T2D in Koreans; 2) to investigate the prospective association between the SNPs and the risk of T2D; 3) to study the interaction effect between dietary iron intake and the SNPs, prospectively.

## Materials and methods

### Study dataset

The study dataset was an ongoing community-based cohort included in the Korean Genome and Epidemiology Study (KoGES_Ansan and Ansung Study). Study participants consisted of 10,038 men and women recruited from the city of Ansan (5,020 participants), located 25 miles southwest of the capital city, Seoul, and the city of Ansung (5,018 participants), located 47 miles south of Seoul. Ansan is an industrialized community and Ansung is a rural area. The baseline study was conducted between June 18, 2001 and January 29, 2003 and follow-up examinations were conducted biennially with a scheduled site visit. The details of the study design and procedures are described in a previous report [16].

DNA samples were obtained from 10,004 participants in 2 cohorts. Through the sample and SNP quality controls, a total of 8,842 individuals were included in the data analysis [17]. We additionally excluded participants who did not answer to history taking in medicine (*n* 146), reported coronary artery disease (CAD), stroke, or cancer (*n* 354), did not complete the food frequency questionnaire (*n* 248), or reported an implausible energy intake (<500 kcal/d or >4,000 kcal/d), (*n* 159).

**Fig 1:**
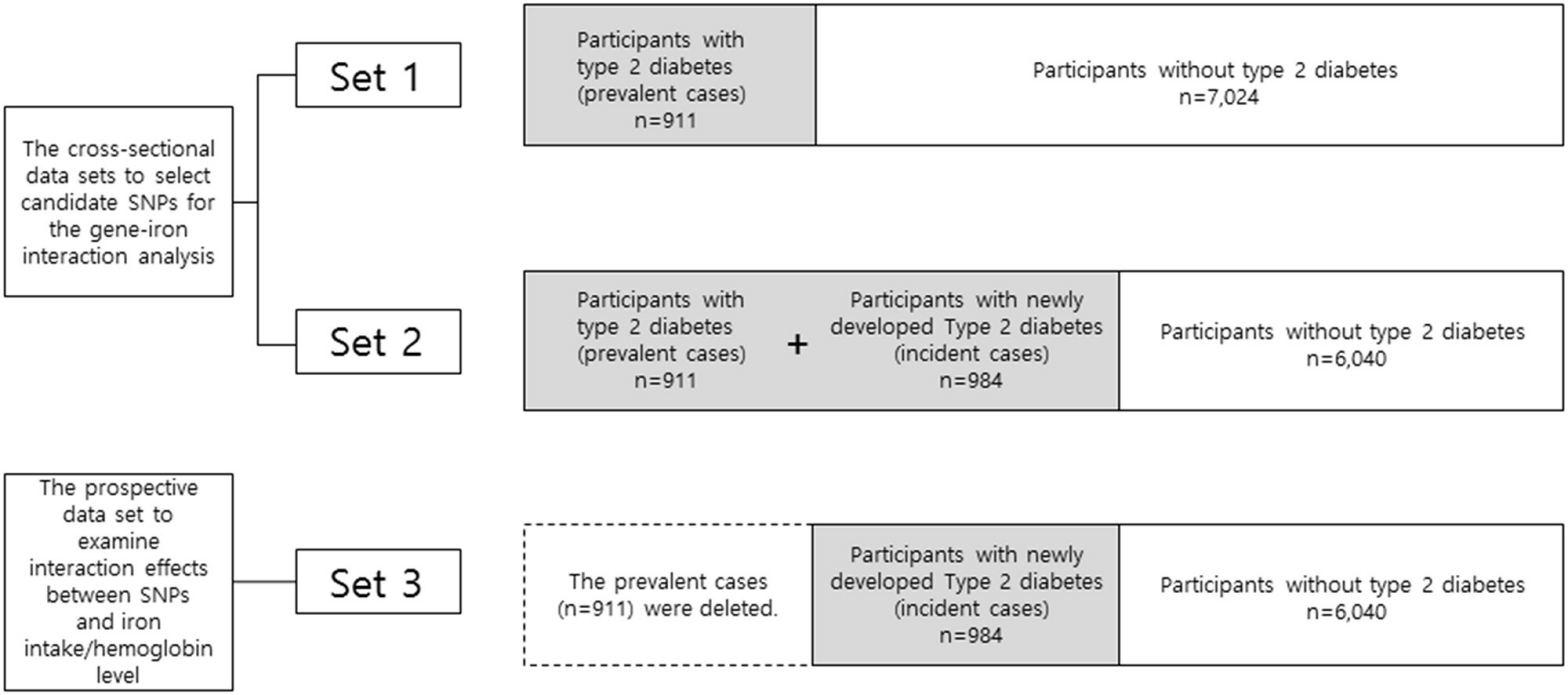
The data sets used for a two-step approach.

The gene-iron interaction was conducted by a two-step approach. Three data sets were used (Fig 1). The first step was a screening step using data set 1 and set 2 and the second was a gene-iron interaction step using data set 3. To select candidate SNPs for the gene-iron interaction analysis, association analyses between genes and T2D were conducted in the first step. This step was composed of two data sets for a genome-wide association (GWA) analysis: one data set (set 1) consisted of 7,935 participants (911 prevalent cases and 7,024 non-cases (3811 men and 4124 women), and the second dataset (set 2) consisted of 1895 cases (911 prevalent cases and 984 incident cases) and 6,040 non-cases. We then prospectively examined whether the SNPs identified in step 1 were associated with T2D and interacted with iron to influence the risk of T2D, after excluding the prevalent cases (*n* 911) of set 1 (set 3, *n* 7,024) in the second step. All procedures involving human subjects were approved by the Institutional Review Board of Kyung Hee University in accordance with the Declaration of Helsinki. Written informed consent was obtained from all subjects.

### General characteristics, anthropometrics, and biochemical variables

Participants completed comprehensive health examinations and interviewer-administered questionnaires, which contained questions on demographic information, medical history, family history, and lifestyles. Interviewers were trained how to administer the questionnaire with a standardized manual and all procedures were conducted with a standardized protocol. Participants were weighed in kilograms to the nearest 0.1 kg and height was measured within 0.1 cm without shoes. Body mass index (BMI) was calculated as weight (kg)/height (m^2^). Waist circumference (WC) was measured at the narrowest part between the lowest rib and the iliac crest to the nearest 0.1 cm. Blood pressure was measured in the supine position with mercury sphygmomanometers. red to a central laboratory (Seoul Clinical Laboratories, Seoul, Korea) for measuring serum concentrations of glucose, triglyceride, total cholesterol, HDL-cholesterol, high-sensitivity C-reactive protein (hsCRP), and for measuring the whole blood concentration of hemoglobin. Fasting glucose, triglyceride, total cholesterol, and HDL-cholesterol were measured using an autoanalyzer and hsCRP was measured by immunoradiometric assay (ADVIA1650; Siemens, NY, USA). Hemoglobin was measured by an autoanalyzer (ADVIA120; Siemens, NY, USA).

Although hepcidin peptide hormone and serum ferritin may be more valid biomarkers of iron status [18], there was unfortunately no data on these markers in the Ansung-Ansan cohort. Since it has been reported that serum hemoglobin level was associated with ferritin, hepcidin, and the risk of T2D [9, 19], we used hemoglobin levels as a surrogate biomarker of iron status [20, 21]. The central 95 percentile intervals for hemoglobin were 13.3–16.7 g/dL in men and 11.6–14.2 g/dL in women [22]. We calculated the average hemoglobin level between baseline and before diagnosis of T2D to reflect long-term iron status. We classified participants into 3 groups: <13.3g/dL, 13.3–16.7 g/dL, and >16.7 g/dL in men; <11.6g/dL, 11.6–14.2 g/dL, and >14.2 g/dL in women. Normal average hemoglobin levels were 13.3g/dl for men and 11.6 g/dl for women in this population.

### Dietary measurement

A semi-quantitative food frequency questionnaire (FFQ) with 103 food items was used to estimate nutrient intake. The validity and reproducibility of the FFQ have been previously assessed in detail [23]. Estimation of nutrient intake was done using the seventh edition Food Composition Table of Korea [24]. Nutrient intake was adjusted for total energy intake using the residual method in men and women, separately [25]. Participants were grouped in tertiles by dietary iron intake.

### Genotyping

DNA samples were genotyped on the Affymetrix Genome-Wide Human SNP Array 5.0 using 500 ng of genomic DNA. The Bayesian Robust Linear Modeling using Mahalanobis Distance (BRLMM) Genotyping Algorithm was employed for genotype calling of 500,568 SNPs [26]. Markers with low minor allele frequency (MAF), high missing gene call rate (>5%), significant deviation from Hardy-Weinberg equilibrium (P < 1 x 10^−6^) were excluded, resulting in a total of 352,228 SNPs. Further details regarding the genotype calling and quality control processes are described in a previous study [17].

### Case definition and follow up

The prevalent T2D cases and/or incident T2D cases were used as T2D cases in the analyses for candidate SNPs screening (only prevalent cases were included in set 1; prevalent and incident cases in set 2) and only incident cases were used for interaction analysis (only incident cases in set 3). If a participant reported treatment of T2D by hypoglycemic medications (oral anti-diabetic drugs and/or insulin) and/or satisfied the World Health Organization criteria for T2D (FPG ≥ 7.0 mmol/l (126 mg/dl) and/or plasma glucose 2-h after ingestion of 75g oral glucose load ≥ 11.1mmol/l (200mg/dl) at baseline [27], the participant was defined as a prevalent T2D case. For the diagnosis of incident T2D case, the identical criteria of diagnosis of prevalent T2D cases were applied for self-reported T2D and fasting and oral glucose loading blood glucose levels at each follow-up visit. Participants were followed biennially for 10 years (2001–2010) with repeated measurements using validated questionnaires and health examinations to acquire updated information on lifestyle factors and disease status at each visit. Using this information for 10 years, we ascertained incident cases of T2D (*n* 984).

### Statistical methods

The sex difference in the prevalence of diabetes have been reported from previous studies [28, 29]. Men may have generally higher fasting plasma glucose level than women because men’s hepatic sensitivity to insulin tend to be lower [30]. In addition, since men and women have different life style, all analyses were conducted for men and women, separately.

Association analysis between SNPs and T2D was conducted using Plink (http://pngu.mgh.harvard.edu/~purcell/plink2/) and SAS software (version 9.3; SAS Institute, Cary, NC). The gene-iron interaction was conducted by a two-step approach. The first step was a screening step and the second a gene-iron interaction step. In the first step, the association analysis of 352,228 genotyped SNPs and T2D was performed under an additive logistic regression model after adjusting for age, sex, BMI, and residential area using data set 1 and set 2. We screened out SNPs that had either a weak relationship to type 2 diabetes (*P*≥5x10^-6^). A total of 8 SNPs in set 1 and 2 SNPs in set 2 passed the criteria (*P*<5x10^-6^). For 8 SNPs on CDKAL1 in set 1, we additionally examined the linkage disequilibrium (LD) analyses. Eight SNPs were located on identical genes and loci and showed strong LDs of r^2^ = 0.90 (rs7747752), 0.86 (rs2328549), 0.90 (rs7767391), 0.63 (rs9460546), 0.63 (rs7754840), 0.63 (rs6456368), and 0.64 (rs10946398), with rs9465871 having the lowest *P* value. Therefore, only rs9465871 was included in the following analysis. Finally, 3 SNPs were selected (Table 1) for gene-iron interaction analysis: one SNP was selected from set 1 [(rs9465871 on CDKAL1 (CDK5 regulatory subunit associated protein 1-like 1)] and two SNPs [rs10761745 on JMJD1C (jumonji domain containing 1C) and rs163177 on KCNQ1 (potassium voltage-gated channel KQT-like subfamily, member 1)] were adopted from set 2. While the association between CDKAL1 and T2D has been well known in GWAS study using prevalent cases [31, 32] and also KCNQ1 was identified as a T2D susceptibility gene in Asian and European populations [33, 34], there was no evidence on the association between JMJD1C and T2D.

**Table 1:** Description of SNPs related to type 2 diabetes*.

SNP	Chromosome	Position	Locus	Gene symbol	Location	Minor allele	MAF	RAF	Set 1		Set 2		Set 3	
									OR	P	OR	P	HR	P
rs9465871	6	20825234	6p22.3	CDKAL1	intron	T	0.45	0.55	0.76	6.06 x10^-8^	0.85	1.85 x10^-5^	0.94	1.83 x 10^−1^
rs10761745	10	64771077	10q21.2	JMJD1C	intron	G	0.38	0.62	0.88	1.94 x 10^−2^	0.83	4.67 x10^-6^	0.82	5.30 x 10^−5^
rs163177	11	2794989	11p15.5	KCNQ1	intron	C	0.43	0.43	1.18	9.65 x 10^−4^	1.21	1.24 x10^-6^	1.17	6.25 x 10^−4^

MAF, minor allele frequency; RAF, risk allele frequency; SNP, single nucleotide polymorphism.

*The SNPs were selected by a logistic regression model with data sets 1 and 2 and were re-evaluated by a Cox proportional hazard model (set 3).

In data set 3, for each participant, person-time were calculated from baseline survey to the date of T2D diagnosis, loss to follow-up, or the end of final survey in 2010, whichever came first. A Cox proportional hazard model was applied to model time to incident T2D events and hazard ratios (HRs) and 95% confidence intervals (CIs) were obtained. The prospective association between these 3 SNPs finally selected in the screening step and T2D incidence and the interaction between SNPs and iron status on T2D were confirmed and examined using a Cox proportional hazard model (Table 1).

In the second step, all analyses were separately performed for men and women in set 3. Descriptive statistics on the baseline characteristics for men and women are shown in S1 Table. Potential confounders were evaluated using an age-adjusted general linear model and only variables exhibiting significant linear trends across tertiles of iron intake or hemoglobin levels groups were included in multivariable analyses as confounders.

The validity of the proportional hazards assumption was assessed using two different approaches. First, the assumption was graphically assessed by log-log plots. Second, the proportional hazards assumption was examined using a time-dependent covariate by including an interaction term between the main exposure (iron intake) and log of follow-up time. The time-dependent covariate was not statistically significant (*P* = 0.5744 in men; *P* = 0.1412 in women) and the proportionality was reasonable. The interaction was assessed by comparing nested models with and without interaction terms using the likelihood ratio test. Based on Bonferroni correction, statistical significance was set at 0.0083 for multiple testing (6 tests; 3 SNPs for men and women). Additionally, a false discovery rate (FDR) correction was used with a threshold of < 0.1 for statistical significance [35, 36].

## Results

Table 1 shows the candidate SNPs from the screening step. Among the three SNPs from sets 1 and 2, CDKAL1 was not associated with incident risk of T2D, but the JMJD1C polymorphism in the rs10761745 region (C>G) (HR = 0.82 and *P* = 5.30x10^-5^) and the KCNQ1 polymorphism in the rs163177 region (T>C) were significantly associated with an increased risk of T2D (HR = 1.17 and *P* = 6.25x10^-4^).

The general characteristics of the participants by dietary iron intake tertiles and average hemoglobin level groups are shown in Table 2. Men in the highest tertile of iron intakes tended to be slightly younger, unlikely to be former drinker and current smoker, and to have lower carbohydrate intake comparing with those in the lowest tertile, while they tended to have higher BMI and WC, to have higher intakes of protein, fat, zinc, vitamin C, tea, and meat, to be residents in Ansan, and to be more likely current drinker, regular exerciser, and dietary supplement user. In women, those with a higher iron intake tended to be younger, had a lower carbohydrate intake, were more likely to be residents of Ansan, had a higher education level, were dietary supplementary users, and had a higher intake of protein, fat, zinc, vitamin C, tea, and meat. In comparison with average hemoglobin level groups, we noted that men in the highest average hemoglobin group tended to be younger. Compared to the lowest average hemoglobin group, men in the highest group had a high school or above education and were more likely to be current smokers. Women in the highest average hemoglobin group tended to be older, lived in an urban area, and were more likely to be a current drinker. Compared to women in the lowest average hemoglobin group, the highest group had a lower proportion of former smokers and consumed more energy, carbohydrates, protein, and tea. BMI and WC increased across the average hemoglobin level groups in men and women. Variables showing significant trends across tertiles of iron intake or hemoglobin level groups in Table 2 were adjusted as potential confounders in Tables 3 and 4.

**Table 2:** Age-adjusted baseline characteristics of the study subjects based on iron intake group and hemoglobin level*.

	Tertile of iron intake (mg/d)						P for linear trend	Average hemoglobin levels (g/dL)						P for linear trend
	Low		Medium		High			<13.3/11.6		13.3–16.7/11.6–14.2		>16.7/14.2		
Men, n	1,108		1,109		1,109			215		3,045		66		
Median (min, max)	7.9 (4.0, 9.2)		10.2 (9.2, 11.3)		12.5 (11.3, 28.8)			12.9 (6.4, 13.3)		14.9 (13.3, 16.7)		17.1 (16.7, 19.5)		
	Mean	SD	Mean	SD	Mean	SD		Mean	SD	Mean	SD	Mean	SD	
Age (years)	51.9	8.9	50.3	8.4	50.6	8.3	0.0004	57.4	8.8	50.5	8.4	48.3	6.1	<0.0001
BMI (kg/m^2^)	24.0	0.1	24.1	0.1	24.4	0.1	0.0004	22.7	0.2	24.2	0.1	25.5	0.3	<0.0001
Waist circumference (cm)	82.8	0.2	82.9	0.2	83.9	0.2	0.0005	79.8	0.5	83.4	0.1	86.6	0.9	<0.0001
Daily dietary intake														
Total energy intake (kcal)	1,987.4	16.1	1,972.8	16.1	2,020.9	16.1	0.1383	1960.9	37.3	1,995.5	9.7	2,018.3	66.0	0.7905
Total energy intake (kJ)	8,315.3	16.1	8,254. 2	16.1	8,455.4	16.1	0.1383	8,204.4	37.3	8,349.2	9.7	8,444.6	66.0	0.7905
Carbohydrate (g)	354.0	2.6	338.7	2.6	340.5	2.6	0.0003	343.3	6.1	344.4	1.6	348.0	10.8	0.6555
Protein (g)	61.2	0.7	67.9	0.7	76.7	0.7	<0.0001	66.3	1.7	68.7	0.4	70.1	3.0	0.9297
Fat (g)	32.7	0.5	36.1	0.5	37.7	0.5	<0.0001	33.2	1.2	35.7	0.3	35.6	2.2	0.9184
Zn (μg)	7.3	0.1	8.3	0.1	9.4	0.1	<0.0001	8.0	0.1	8.3	0.04	8.4	0.3	0.0664
Vitamin C (mg)	76.7	1.4	104.5	1.4	139.8	1.4	<0.0001	106.8	3.8	107.0	1.0	107.4	6.7	0.5104
Tea intake (g)	26.2	2.0	41.5	2.0	57.5	2.0	<0.0001	40.5	4.7	41.7	1.2	45.6	8.4	0.3831
Meat intake (g)	53.6	1.5	59.2	1.5	62.5	1.5	<0.0001	50.0	3.6	58.9	0.9	65.4	6.3	0.1406
Residential area-Ansan (%)	48.8		64.4		67.3		<0.0001	45.9		60.9		74.3		0.6914
Education, high school or beyond (%)	54.9		61.7		63.1		<0.0001	53.1		60.5		55.7		0.0024
Drinking status														
Former drinker (%)	11.2		8.9		7.8		0.0052	16.2		8.8		9.8		0.0617
Current drinker (%)	70.2		71.8		74.6		0.0198	71.7		72.3		71.3		0.4359
Smoking status														
Former smoker (%)	28.4		32.3		30.8		0.2297	33.7		30.4		23.1		0.8641
Current smoker (%)	54.3		47.3		48.0		0.0033	51.7		49.6		55.4		0.0032
Regular exercise (%)	9.7		13.7		16.3		<0.0001	5.5		13.9		8.9		0.5728
Dietary supplement user (%)	12.6		14.1		18.3		0.0005	11.7		15.2		13.0		0.6065
Women, n	1,232		1,233		1,233			452		3,147		99		
Median (min, max)	7.5 (2.9, 8.7)		9.8 (8.7, 10.7)		12.1 (10.7, 35.3)			11.1 (3.7, 11.6)		12.8 (11.6, 14.2)		14.5 (14.2, 17.5)		
	Mean	SD	Mean	SD	Mean	SD		Mean	SD	Mean	SD	Mean	SD	
Age (years)	53.2	9.1	51.2	8.7	51.1	8.7	<0.0001	49.2	9.2	52.1	8.7	54.5	8.6	<0.0001
BMI (kg/m^2^)	24.6	0.1	24.8	0.1	24.8	0.1	0.0697	24.1	0.2	24.8	0.1	26.2	0.3	0.0107
Waist circumference (cm)	81.1	0.3	80.6	0.3	80.8	0.3	0.3697	79.3	0.4	80.9	0.2	85.2	0.9	0.0002
Daily dietary intake														
Total energy intake (kcal)	1,830.4	16.0	1,853.1	16.0	1,824.1	16.0	0.7608	1,793.5	26.5	1,842.5	10.0	1,819.1	56.3	0.0067
Total energy intake (kJ)	7,658.4	16.0	7,753.4	16.0	7,632.0	16.0	0.7608	7,504.0	26.5	7,709.0	10.0	7,611.1	56.3	0.0067
Carbohydrate (g)	338.8	2.8	330.4	2.8	319.0	2.8	<0.0001	323.5	4.7	330.3	1.8	326.0	10.1	0.0083
Protein (g)	54.5	0.6	62.3	0.6	67.7	0.6	<0.0001	59.4	1.1	61.8	0.4	61.3	2.3	0.0086
Fat (g)	25.3	0.4	29.5	0.4	30.3	0.4	<0.0001	27.2	0.7	28.6	0.3	28.4	1.5	0.0534
Zn (μg)	7.1	0.1	8.0	0.1	9.0	0.1	<0.0001	7.8	0.1	8.0	0.04	8.3	0.2	0.0888
Vitamin C (mg)	88.8	1.8	123.4	1.8	167.9	1.8	<0.0001	124.6	3.3	126.8	1.3	135.5	7.1	0.0769
Tea intake (g)	25.9	2.2	38.7	2.2	50.7	2.2	<0.0001	29.4	3.6	39.7	1.4	40.7	7.7	0.0104
Meat intake (g)	33.2	1.1	38.8	1.1	40.1	1.1	<0.0001	35.1	1.9	37.8	0.7	35.6	4.0	0.7197
Residential area-Ansan (%)	41.0		56.6		56.9		<0.0001	48.7		51.7		59.7		<0.0001
Education, high school or beyond (%)	28.0		34.0		39.5		<0.0001	34.5		33.8		29.9		0.7095
Drinking status														
Former drinker (%)	3.3		2.7		2.9		0.5283	2.5		2.9		8.0		0.4727
Current drinker (%)	27.6		25.9		25.9		0.3346	23.6		26.8		29.1		0.0254
Smoking status														
Former smoker (%)	1.3		1.4		0.9		0.4809	1.0		1.2		0.9		0.0355
Current smoker (%)	3.0		3.3		4.4		0.0647	3.0		3.7		3.0		0.0618
Regular exercise (%)	8.5		15.2		18.8		<0.0001	11.5		14.7		10.2		0.4595
Dietary supplement user (%)	17.3		23.0		26.8		<0.0001	20.9		22.7		20.5		0.7362

BMI, Body mass index

* Values derived by using general linear model analysis adjusted for age.

**Table 3:** HRs (95% CI) of type 2 diabetes according to iron intake& hemoglobin level and interaction between the studied gene polymorphism with iron intakes and hemoglobin level in relation to type 2 diabetes risk*.

	Tertile of iron intake (mg/d)						P	Average hemoglobin levels (g/dL)						P
	Low		Medium		High			<13.3/11.6		13.3–16.7/11.6–14.2		>16.7/14.2		
**Men**, n	1,108		1,109		1,109			215		3,045		66		
Median (min, max)	7.9 (4.0, 9.2)		10.2 (9.2, 11.3)		12.5 (11.3, 28.8)			12.9 (6.4, 13.3)		14.9 (13.3, 16.7)		17.1 (16.7, 19.5)		
No. of cases/person-years	151/6,760		194/6,741		197/6,519			27/1,144		500/18,525		15/351		
HR (95% CI)	1.00†		1.33 (1.05, 1.67)		1.36 (1.04, 1.78)		0.0272‡	1.00		1.11 (0.75, 1.65)		1.54 (0.80, 2.98)		<0.0001
	HR	95% CI	HR	95% CI	HR	95% CI		HR	95% CI	HR	95% CI	HR	95% CI	
rs9465871 (CDKAL1)														
TT	1.00§		1.01	0.59, 1.72	1.28	0.78, 2.12	0.9122¶	1.00		1.15	0.42, 3.13	2.97	0.74, 11.93	0.5771
CT	1.14	0.74, 1.75	1.54	1.01, 2.34	1.50	0.96, 2.35		1.10	0.36, 3.39	1.47	0.55, 3.95	1.73	0.50, 5.92	
CC (wild type)	1.07	0.67, 1.71	1.50	0.95, 2.36	1.57	0.98, 2.51		1.62	0.51, 5.18	1.38	0.51, 3.73	1.54	0.34, 6.90	
rs10761745 (JMJD1C)														
GG	1.00		1.06	0.53, 2.11	1.62	0.85, 3.08	0.6592	1.00		0.49	0.20, 1.23	1.85	0.21, 15.93	0.1910
CG	1.24	0.72, 2.14	1.65	0.96, 2.82	1.52	0.86, 2.66		0.64	0.23, 1.76	0.61	0.25, 1.49	0.80	0.25, 2.54	
CC (wild type)	1.52	0.88, 2.63	2.08	1.21, 3.57	2.17	1.25, 3.79		0.44	0.14, 1.38	0.84	0.34, 2.04	1.07	0.33, 3.53	
rs163177 (KCNQ1)														
TT (wild type)	1.00		1.58	1.04, 2.39	1.41	0.91, 2.19	0.0193	1.00		1.10	0.49, 2.51	2.29	0.76, 6.85	0.4432
TC	1.11	0.75, 1.66	1.66	1.13, 2.45	1.98	1.32, 2.98	(0.1158)**	1.16	0.45, 3.03	1.32	0.58, 2.98	0.98	0.28, 3.51	
CC	2.31	1.50, 3.57	1.89	1.20, 2.96	1.78	1.08, 2.91		1.41	0.47, 4.19	1.65	0.72, 3.77	4.72	1.17, 19.10	
**Women**, n	1232		1233		1233			452		3147		99		
Median (min, max)	7.5 (2.9, 8.7)		9.8 (8.7, 10.7)		12.1 (10.7, 35.3)			11.1 (3.7, 11.6)		12.8 (11.6, 14.2)		14.5 (14.2, 17.5)		
No. of cases/person-years	114/7528		156/7520		172/7467			41/2726		377/19225		24/566		
HR (95% CI)	1.00		1.32 (1.02, 1.72)		1.59 (1.18, 2.14)		0.0023	1.00		1.09 (0.79, 1.51)		1.76 (1.06, 2.94)		<0.0001
	HR	95% CI	HR	95% CI	HR	95% CI		HR	95% CI	HR	95% CI	HR	95% CI	
rs9465871 (CDKAL1)														
TT	1.00		1.35	0.77, 2.39	1.80	1.02, 3.19	0.4636	1.00		1.11	0.56, 2.22	1.09	0.24, 5.09	0.6282
CT	1.02	0.61, 1.71	1.54	0.94, 2.54	1.48	0.88, 2.48		0.92	0.42, 2.06	1.07	0.55, 2.10	1.51	0.64, 3.54	
CC (wild type)	1.19	0.69, 2.06	1.26	0.73, 2.17	1.92	1.12, 3.31		1.08	0.47, 2.49	1.05	0.53, 2.08	2.71	1.07, 6.86	
rs10761745 (JMJD1C)														
GG	1.00		2.04	0.94, 4.46	1.96	0.85, 4.52	0.3027	1.00		1.16	0.36, 3.74	1.16	0.19, 7.04	0.0417
CG	1.75	0.87, 3.54	1.97	0.97, 3.98	2.92	1.44, 5.91		1.28	0.38, 4.31	1.49	0.48, 4.68	1.24	0.32, 4.80	(0.2502)
CC (wild type)	1.83	0.90, 3.74	2.70	1.34, 5.45	2.48	1.20, 5.10		1.48	0.43, 5.05	1.50	0.48, 4.70	5.09	1.47, 17.64	
rs163177 (KCNQ1)														
TT (wild type)	1.00		1.39	0.89, 2.17	1.45	0.90, 2.35	0.9232	1.00		0.90	0.52, 1.58	2.00	0.88, 4.54	0.3823
TC	1.11	0.72, 1.71	1.47	0.97, 2.24	1.83	1.19, 2.81		1.11	0.57, 2.15	1.13	0.65, 1.94	1.65	0.75, 3.65	
CC	1.29	0.77, 2.18	1.54	0.94, 2.51	1.72	1.02, 2.92		0.44	0.13, 1.54	1.21	0.69, 2.14	1.36	0.39, 4.76	

* Values are HR (95% CI) determined using a Cox proportional hazard model.

^†^HRs were calculated for iron intake after adjusting for age, residential area, education, drinking status, smoking status, regular exercise, dietary supplement use, WC, and carbohydrate, zinc, vitamin C, tea, and meat intake in men and for age, residential area, education, and carbohydrate, zinc, vitamin C, tea, and meat intake in women.

^‡^ P for linear trend.

^§^ HRs were calculated for iron intake after adjusting for age, smoking status, education, and WC in men and for age, residential area, drinking status, smoking status, WC, energy intake, and tea intake in women.

^¶^ P for interaction.

** FDR, false discovery rate.

**Table 4:** HRs (95% CI) of type 2 diabetes according to iron intake and hemoglobin level and interaction between the studied gene polymorphisms with iron intake and hemoglobin level in relation to type 2 diabetes risk among participants with normal inflammation (hsCRP level of ≥1.0 mg/dL) and without anemia at baseline*.

	Tertile of iron intake (mg/d)						P	Average hemoglobin levels (g/dL)				P
	Low		Medium		High			13.3–16.7/11.6–14.2		>16.7/14.2		
**Men**, n	982		1,024		1,000			2,884		66		
Median (min, max)	8.0 (4.0, 9.2)		10.2 (9.2, 11.3)		12.5 (11.3, 27.9)			14.9 (13.3, 16.7)		17.1 (16.7, 19.5)		
No. of cases/person-years	135/6,058		178/6,251		184/5,911			480/17,490		15/351		
HR (95% CI)	1.00†		1.31 (1.03, 1.68)		1.39 (1.04, 1.85)		0.0268‡	1.00		1.38 (0.81, 2.35)		<0.0001
	HR	95% CI	HR	95% CI	HR	95% CI		HR	95% CI	HR	95% CI	
rs9465871 (CDKAL1)												
TT	1.00§		0.96	0.55, 1.65	1.22	0.73, 2.06	0.8766¶	1.00		2.56	0.94, 6.99	0.4149
CT	1.10	0.71, 1.72	1.47	0.96, 2.26	1.47	0.93, 2.33		1.27	0.998, 1.62	1.48	0.68, 3.19	
CC (wild type)	0.98	0.60, 1.60	1.41	0.88, 2.25	1.51	0.93, 2.46		1.19	0.91, 1.55	1.33	0.42, 4.22	
rs10761745 (JMJD1C)												
GG	1.00		0.88	0.41, 1.88	1.62	0.82, 3.22	0.3637	1.00		3.98	0.55, 28.85	0.5538
CG	1.27	0.71, 2.27	1.70	0.96, 3.02	1.50	0.82, 2.73		1.30	0.96, 1.77	1.70	0.77, 3.75	
CC (wild type)	1.55	0.87, 2.78	2.12	1.19, 3.77	2.32	1.29, 4.18		1.80	1.33, 2.44	2.24	0.96, 5.22	
rs163177 (KCNQ1)												
TT (wild type)	1.00		1.55	1.01, 2.38	1.35	0.85, 2.14	0.0219	1.00		2.03	0.95, 4.35	0.1671
TC	1.06	0.70, 1.61	1.58	1.06, 2.36	1.96	1.29, 2.99	(0.1314)**	1.18	0.96, 1.45	0.88	0.33, 2.38	
CC	2.32	1.48, 3.64	1.84	1.15, 2.95	1.82	1.10, 3.03		1.48	1.15, 1.91	4.16	1.32, 13.12	
**Women**, n	1037		1040		1042			2911		96		
Median (min, max)	7.5 (2.9, 8.7)		9.7 (8.7, 10.7)		12.1 (10.7, 35.3)			12.9 (11.6, 14.2)		14.5 (14.2, 17.5)		
No. of cases/person-years	104/6297		141/6286		149/6301			365/17548		24/549		
HR (95% CI)	1.00		1.35 (1.03, 1.79)		1.57 (1.14, 2.15)		0.0058	1.00		1.60 (1.05, 2.43)		<0.0001
	HR	95% CI	HR	95% CI	HR	95% CI		HR	95% CI	HR	95% CI	
rs9465871 (CDKAL1)												
TT	1.00		1.23	0.69, 2.19	1.50	0.83, 2.72	0.5139	1.00		0.93	0.23, 3.79	0.3325
CT	0.90	0.53, 1.50	1.43	0.86, 2.37	1.33	0.78, 2.27		0.94	0.72, 1.23	1.33	0.74, 2.40	
CC (wild type)	0.99	0.56, 1.75	1.20	0.68, 2.09	1.76	1.01, 3.08		0.94	0.70, 1.26	2.29	1.15, 4.59	
rs10761745 (JMJD1C)												
GG	1.00		2.49	1.10, 5.64	2.27	0.94, 5.48	0.0493	1.00		0.93	0.22, 3.89	0.0077
CG	1.75	0.83, 3.70	2.02	0.95, 4.27	3.22	1.52, 6.81	(0.1479)	1.22	0.87, 1.70	1.01	0.45, 2.24	(0.0462)
CC (wild type)	1.98	0.93, 4.21	2.95	1.40, 6.20	2.25	1.04, 4.89		1.19	0.84, 1.68	3.94	2.19, 7.11	
rs163177 (KCNQ1)												
TT (wild type)	1.00		1.51	0.93, 2.43	1.53	0.92, 2.57	0.9150	1.00		2.33	1.21, 4.49	0.3856
TC	1.12	0.71, 1.78	1.60	1.03, 2.50	1.91	1.21, 3.02		1.27	0.99, 1.62	1.77	0.95, 3.32	
CC	1.43	0.84, 2.45	1.75	1.05, 2.92	1.91	1.09, 3.33		1.38	1.02, 1.85	1.47	0.46, 4.63	

* Values are HR (95% CI) determined using a Cox proportional hazard model.

^†^ HRs were calculated for iron intake after adjusting for age, residential area, education, drinking status, smoking status, regular exercise, dietary supplement use, WC, and carbohydrate, zinc, vitamin C, tea, and meat intake in men and for age, residential area, education, and carbohydrate, zinc, vitamin C, tea, and meat intake in women.

^‡^ P for linear trend.

^§^ HRs were calculated for iron intake after adjusting for age, smoking status, education, and WC in men and for age, residential area, drinking status, smoking status, WC, energy intake, and tea intake in women. There were no incident cases in participants with an average hemoglobin level of <13.3/16.7g/dL (men/women) and homozygote major allele carriers; therefore, participants with an average hemoglobin level of <13.3/16.7g/dL (men/women) were excluded from analysis.

^¶^ P for interaction.

** FDR, false discovery rate.

Table 3 shows the multivariable-adjusted HRs for T2D by tertiles of iron intake and average hemoglobin level groups. Dietary iron intake was significantly positively associated with T2D after adjusting for age, residential area, WC, education, drinking status, smoking status, regular exercise, dietary supplement use, and carbohydrate, zinc, vitamin C, tea, and meat intake in men; a similar trend was found in women after adjusting for age, residential area, education, regular exercise, dietary supplement use, and carbohydrate, zinc, vitamin C, tea, and meat intake (3rd vs. 1st tertile: HR = 1.36, 95% CI = 1.04–1.78, *P* = 0.0272 for trend in men; HR = 1.59, 95% CI = 1.18–2.14, and *P* = 0.0023 for trend in women). Average hemoglobin level was significantly positively associated with T2D after adjusting for age, residential area, smoking status, WC, and energy and tea intake in women (highest group vs. lowest group: HR = 1.76, 95% CI = 1.06–2.94, and *P* for trend<0.0001). A linear trend of the risk of T2D was identified in men (*P* for trend<0.0001). We then evaluated whether genetic polymorphisms of CDKAL1 (rs9465871), JMJD1C (rs10761745), and KCNQ1 (rs163177) and iron interacted in the development of T2D, where the minor alleles of CDKAL1 and JMJD1C were used as references to calculate HR (Table 3). We did not find significant interaction effects on the risk of T2D between the 3SNPs with iron intake and average hemoglobin levels at Bonferroni and FDR significance levels. However, using a significance threshold of *P*<0.05, we found that rs163177 (KCNQ1) interacted with iron intake in men (*P* for interaction = 0.0193, FDR = 0.1158) and rs10761745 (JMJD1C) interacted with average hemoglobin levels in women (*P* for interaction = 0.0417, FDR = 0.2502).

Table 4 shows the prospective interaction effects between gene and iron status after excluding subjects with hsCRP≥1.0 mg/dL or average hemoglobin level <13.3/11.6 g/dL (men/women) in order to reduce confounding effects related to inflammation and anemia. In women, rs10761745 (JMJD1C) had significant average hemoglobin level-dependent effects on the risk of T2D (*P* for interaction = 0.0077, FDR = 0.0462). Using a significance threshold of *P*<0.05, iron intake significantly interacted with rs10761745 (JMJD1C) in women (*P* for interaction = 0.0493, FDR = 0.1479) and rs163177 (KCNQ1) in men.

## Discussion

In the present study, we identified that loci JMJD1C at 10q21.2 and KCNQ1 at 11p15.5 were prospectively significantly associated with the risk of T2D. Iron intake was significantly associated with the risk of T2D in men and women and average hemoglobin level also showed a significant linear trend with T2D in men and women. JMJD1C interacted with average hemoglobin levels in women on the risk of T2D after excluding subjects with hsCRP≥ 1.0 mg/dL or an average Hgb level <13.3/11.6 g/dL (men/women). Interestingly, in our study, we found no significant association between CDKAL1 and T2D risk.

The association between higher total iron intake and an increased risk of T2D among women in the present study disagrees with a meta-analysis that established that non-heme iron and total iron intake were not associated with a higher risk of T2D [7]. We did not find any association between iron intake from animal sources and T2D in additional analysis (data not shown). However, this meta-analysis included 11 prospective studies consisting of 10 Western countries (including USA, Finland, and UK) and 1 Asian country (China). It was likely that dietary differences contributed to this discrepancy. In Korea, the major source of iron is rice and vegetables [37], mostly containing non-heme iron. Total iron intake was positively correlated with serum ferritin [38] and green leafy vegetables were also a determinant of hemoglobin level [39]. A Chinese study showed that subjects who acquired most of their iron from non-heme iron sources in rural areas had adequate ferritin levels[40]. Although heme iron has a higher bioavailability, non-heme iron and heme iron are not distinguishable after absorption [6]. Furthermore, the adaptive HFE variants in Asians likely contributed to elevated iron absorption [41]. Therefore, total iron intake can influence the risk of T2D even when non-heme iron was the major source of intake.

In the present study, a SNP in the 10q21 region (rs10761745) was significantly associated with a lower risk of T2D. Among participants with no pathologic inflammation (hsCRP levels ≥1.0 mg/dL) and without anemia at baseline, the interaction effect between the SNP in the 10q21 region and average hemoglobin level was identified in women. Four genes (JMJD1C, EGR2, NRBF2, and REEP3) are located in the 10q21 region. Although the function of JMJD1C is unclear until now, of four genes, JMJD1C may be the most plausible candidate gene as a type 2 diabetes susceptibility gene, because previous studies have reported that JMJD1C may be associated with serum sex hormone-binding globulin (SHBG) levels and serum androgen levels [42, 43] that may influence pathogenesis of type 2 diabetes [44]. To date, no study has reported on the interaction effects of genetic variations of CDKAL1, JMJD1C, or KCNQ1 with iron intake on the risk of T2D. A potential interaction between HFE genetic variation and heme iron intake has been previously suggested [14], but no SNPs have been found in the heme iron metabolic pathway showing significant interactions with heme iron intake [15]. SNPs at locus JMJD1C have been reported to have a negative correlation with lipoprotein level [45] and a positive correlation with plasma alkaline phosphate [46]. A SNP (rs7910927) at locus JMJD1C was associated with increased serum SHBG levels in men and women [42]. In LD analysis, rs10761745 identified in the present study and rs7910927 [42] at JMJD1C were in high LD (D′ = 1.00). Thus, rs10761745 may be involved in regulating levels of SHBG. Higher SHBG means lower bioavailable testosterone (free and albumin-bound testosterones) [47], which may result in reduced hepcidin suppression [48]. Androgens may indirectly contribute to erythropoiesis through the suppression of hepcidin, which is a key peptide hormone regulating iron homeostasis [48, 49]. This may result in increased iron export into blood from enterocytes, thereby changing body iron stores. Hence, reduced hepcidin suppression may cause decreased dietary iron absorption and iron release from macrophages. Therefore, decreased dietary iron absorption and iron release from macrophages may help reduce the risk of T2D [6].

Genetic variation of KCNQ1 (rs163177) was associated with development of T2D in this study. Indeed, the major allele (C) of rs2237892 in KCNQ1, which was in high linkage disequilibrium (LD) with rs163177 (D′ = 1.00), was significantly associated an increased risk of T2D [50]. In a meta-analysis, the summary per-allele odds ratio of the C variant of rs2237892 for T2D was 1.32 (95% CI: 1.26–1.38) [50]. This could be explained by the fact that KCNQ1 encodes the pore-forming subunit of a voltage-gated K+ channel (KvLQT1) and is also expressed in pancreatic islets; thus, variants of KCNQ1 may be in connection with insulin section [50]. On the contrary, KCNQ1 mutation may reduce gastric acid secretion, which could cause decreased absorption of ferric iron [51] and thereby reduce the risk of T2D [6, 51]. Although KCNQ1 may be related to iron metabolism, it is likely that its beneficial influence on T2D is weaker than its harmful effects.

In this study, we identified that there was cross-sectional association between variants near CDKAL1 and T2D, but the variants were not associated with incidence of T2D. A GWAS study in a Chinese Han Population has reported that the four CDKAL1 SNPs (rs7754840, rs10946398, rs7756992, and rs9465871) were cross-sectionally associated with T2D (range of odds ratio: 1.38–1.49; *P* < 1.9 x 10^−5^) [52].

The lack of an association between CDKAL1 and the incidence of T2D is not in line with previous cross-sectional observations [52, 53], but does agree with a prospective study in the U.S. [54]. The definite function of this gene is not well known. However, Genome-wide association studies (GWAS) have reported that variants of CDKAL1 was significantly associated with reduced insulin secretion [31, 55]. Therefore, the function of CDKAL1 may be related to *β*-cell function. We could not assert why CDKAL1 was not detected as a SNP affecting T2D incidence in this longitudinal analysis. However, because the present study population was composed of subjects aged 40–69 years in the baseline survey and only non-diabetic subjects were included in the prospective analyses, the influence of genes on the incidence of T2D may be diminished compared to a population including diabetics diagnosed at a younger age. The strengths of the environmental (dietary) and genetic effects may change over the course of a lifetime; genetic effects may decrease with age as environmental effects exert more influence [56]. This finding suggests that SNPs previously found in case-control analyses need to be confirmed in a longitudinal study design and that our results need to be pursued in future large-scale cohort studies.

Our study has some limitations. First, serum ferritin is the most widely used biomarker of body iron status, but it was not examined in the present study. Serum ferritin may be a better indicator of body iron status than iron intake and hemoglobin, but hemoglobin levels are related to serum ferritin and hepcidin [19]. Because 1-year FFQ depends upon the long-term memory of participants, dietary intake accuracy may be lower. In addition, we could not integrate FFQs of the first and third surveys because different questionnaires were used; therefore, we could not calculate average iron intake. Another limitation was that we did not have information on the form of iron (heme/non-heme iron), which is important in determining the bioavailability of iron. Regardless of these limitations, the strength of the present study was a population-based prospective cohort study with genome data and repeated measures of hemoglobin. In addition, the present study provided the first longitudinal evidence on the interaction effect between a SNP of JMJD1C with iron intake and hemoglobin level.

In conclusion, SNPs of JMJD1C at 10q21.2 and KCNQ1 at 11p15.5 were prospectively associated with the risk of T2D. Additionally, CDKAL1 may not be associated with T2D onset over the age of 40. Results from our study confirm that iron intake and hemoglobin levels are associated with a higher risk of T2D. In addition, we found that for women, a SNP of JMJD1C was interacted with average hemoglobin level on the risk of T2D among participants with normal inflammation and without anemia. However, further study on the function of JMJD1C is necessary to confirm these associations for prevention and/or treatment of T2D.

## Supporting information

S1 Table General characteristics of the study participants. (DOCX)
