# Supplementary material for: Interaction of iron status with single nucleotide polymorphisms on incidence of type 2 diabetes
Source: PLoS One. 2017 Apr 13;12(4):e0175681. doi: 10.1371/journal.pone.0175681 (PMC5391066; doi:10.1371/journal.pone.0175681)
Supplement: S1 Table — (DOCX) [file pone.0175681.s001.docx]

# Supporting information

S1 Table. General characteristics of the study participants *

|  | Men | | Women | |
| --- | --- | --- | --- | --- |
| Characteristics | Mean | SD | Mean | SD |
| n | 3,326 | | 3,698 | |
| Age (years) | 50.9 | 8.6 | 51.8 | 8.9 |
| BMI (kg/m^2^) | 24.2 | 2.9 | 24.7 | 3.2 |
| Waist circumference (cm) | 83.2 | 7.5 | 80.8 | 9.6 |
| Blood pressure (mmHg) |  |  |  |  |
| Systolic blood pressure | 116.3 | 16.2 | 115.9 | 19.2 |
| Diastolic blood pressure | 75.9 | 11.2 | 73.3 | 11.8 |
| Triglyceride | 171.9 | 112.6 | 141.3 | 78.1 |
| Total cholesterol (mg/dL) | 191.9 | 35.1 | 189.4 | 34.8 |
| HDL-Cholesterol (mg/dL) | 43.8 | 10 | 46 | 10.1 |
| Fasting blood glucose (mg/dL) | 85.1 | 9.3 | 81.4 | 8 |
| 2-h glucose tolerance test (mg/dL) | 112.9 | 32.5 | 119.3 | 29.2 |
| Residential area-Ansan (%) | 60.2 | | 51.5 | |
| Marital status-married (%)) | 96.1 | | 86.1 | |
| Family history of diabetes (%) | 9.4 | | 11.3 | |
| High school or beyond (%) | 59.9 | | 33.8 | |
| Current drinker (%) | 72.2 | | 26.5 | |
| Current smoker (%) | 49.9 | | 3.6 | |
| Regular exercise (%) | 13.3 | | 14.2 | |
| Hypertension history (%) | 11.5 | | 14.5 | |
| Diabetes history (%) | 1.4 | | 0.7 | |

BMI, Body Mass Index

* Mean values and standard deviations
